# Postcardiotomy Veno-Arterial Extracorporeal Membrane Oxygenation: Does the Cannulation Technique Influence the Outcome?

**DOI:** 10.3389/fcvm.2021.658412

**Published:** 2021-08-09

**Authors:** Nikolaos Kalampokas, Nihat Firat Sipahi, Hug Aubin, Payam Akhyari, Georgi Petrov, Alexander Albert, Ralf Westenfeld, Artur Lichtenberg, Diyar Saeed

**Affiliations:** ^1^Department of Cardiovascular Surgery, University Hospital of Düsseldorf, Düsseldorf, Germany; ^2^Department of Cardiology, Pulmonology, and Vascular Medicine, University Hospital of Düsseldorf, Düsseldorf, Germany; ^3^University Department for Cardiac Surgery, Leipzig Heart Center, Leipzig, Germany

**Keywords:** ECMO, cardiogenic shock, postcardiotomy, cannulation, low cardiac output

## Abstract

**Objectives:** Veno-arterial extracorporeal membrane oxygenation (VA-ECMO) may be cannulated using either central (cannulation of aorta) or peripheral (cannulation of femoral or axillary artery) access. The ideal cannulation approach for postcardiotomy cardiogenic shock (PCS) is still unknown. The aim of this study is to compare the outcome of patients with PCS who were supported with central vs. peripheral cannulation.

**Methods:** This is a single-center retrospective data analysis including all VA-ECMO implantations for PCS from January 2011 to December 2017. The central and peripheral approaches were compared in terms of patient characteristics, intensive care unit (ICU) stay, hospitalization length, adverse event rates, and overall survival.

**Results:** Eighty-six patients met the inclusion criteria. Twenty-eight patients (33%) were cannulated using the central approach, and 58 patients (67%) were cannulated using the peripheral approach. Forty-three patients (50%) received VA-ECMO in the operating room and 43 patients (50%) received VA-ECMO in the ICU. Central VA-ECMO group had higher EuroSCORE II (*p* = 0.007), longer cross-clamp time (*p* = 0.054), higher rate of open chest after the procedure (*p* < 0.001), and higher mortality rate (*p* = 0.02). After propensity score matching, 20 patients in each group were reanalyzed. In the matched groups, no statistically significant differences were observed in the baseline characteristics between the two groups except for a higher rate of open chests in the central ECMO group (*p* = 0.02). However, no significant differences were observed in the outcome and complications between the groups.

**Conclusions:** This study showed that in postcardiotomy patients requiring VA-ECMO support, similar complication rates and outcome were observed regardless of the cannulation strategy.

## Introduction

The application of veno-arterial extracorporeal membrane oxygenation (VA-ECMO) in patients with refractory isolated cardiac or cardiopulmonary failure is increasing (1). Among high-risk patient populations requiring VA-ECMO support include patients with postcardiotomy cardiogenic shock (PCS). As all of these patients have open heart surgery, there are two main modalities to implant the VA-ECMO in these patient populations. These modalities include either central cannulation of the right atrium and ascending aorta or peripheral cannulation, most commonly *via* the femoral vein and artery. Alternative approaches may include the placement of a vascular prosthesis in the ascending aorta for central access (2) or, for peripheral access cannulation of the axillary artery, either directly or through a vascular prosthesis.

The optimal cannulation strategy for VA-ECMO, in terms of survival as well as myocardial recovery, management, and complication rates, remains controversial (3). Despite the considerable numbers of studies on VA-ECMO application, only a few have addressed access-related issues as primary focus in their studies (2, 4, 5). In the largest single-center series to date, Rastan et al. (6) reported no advantage of different cannulation sites by means of survival in 517 patients who required VA-ECMO after cardiac surgery, although there has been a general consensus favoring the peripheral approach (2, 6–8). Meanwhile, a recent study demonstrated that a central approach should be considered as a viable alternative in terms of complication rates (9). Based on the controversies above, we aimed to compare the outcomes of the patients with PCS who were mechanically supported with central vs. peripheral VA-ECMO.

## Materials and Methods

### Definitions and Data Assessment

#### Inclusion and Exclusion Criteria

The inclusion criteria were adult patients (aged > 18 years) who underwent VA-ECMO implantation after elective, urgent, or emergency cardiac surgery either immediately or a few hours after arrival in the intensive care unit (ICU). Exclusion criteria were patients on VA-ECMO prior to index cardiac procedure, patients requiring venovenous (VV)-ECMO, and patients after heart transplantation and/or ventricular assist device implantation. The study protocol was approved from the corresponding institutional ethics committee (Study number: 2018-33-RetroDEuA).

PCS was defined as cardiac failure that results in the inability to wean from cardiopulmonary bypass (CPB) or cardiac failure that appears in early postoperative period under optimized inotropic and vasopressor support. Hypotension, persistent lactatemia as a sign of an end-organ malperfusion, and oliguria were the clinical parameters for the diagnosis, which was supported by an echocardiographic assessment in each patient and hemodynamic monitoring with Swan-Ganz catheterization in most cases.

Central cannulation was defined as the cannulation involving the aorta and right atrium either directly or through percutaneously placed cannula through the femoral veins. Peripheral cannulation was defined as the cannulation of the femoral or subclavian artery and femoral vein.

Bleeding was defined as any bleeding requiring reoperation. Peripheral vascular (PV) complication was defined as any extremity complication involving the vascular access (excluding groin infection). Notably, all patients with peripheral VA-ECMO cannulation technique were supported with distal leg perfusion catheter to avoid limb ischemia. Postoperative gastrointestinal (GI) complication was defined as postoperative new-onset GI bleeding or ischemia requiring surgery. Postoperative neurological injury was defined as any neurological complication including transient ischemic attack, non-disabling or disabling stroke, and global brain ischemia. Postoperative liver failure was defined as an acute increase in serum aspartate aminotransferase (AST), alanine aminotransferase (ALT), and bilirubin.

The following data were assessed: patient characteristics, type of the cardiac procedure, urgent or emergency procedure, cross-clamp time, CPB time, EuroSCORE II, VA-ECMO support duration, place of VA-ECMO implantation (intraoperative or in the ICU), and rate of chest being left open at the primary surgery. Furthermore, the following postimplantation data were documented: chest tube output in the first 24 h after implantation, bleeding requiring a reoperation, number of red blood cell (RBC) units given, new onset of renal dialysis, postoperative neurological injury, liver failure, and GI and PV complications. Weaning and explantation rate from ECMO, duration of ICU stay, and mortality rate after ECMO implantation were documented and compared between both groups.

### Statistical Analysis

Using the SPSS statistical package and in order to test the effect of the ICU stay, hospitalization length, adverse event rates, and overall survival on the two groups (central and peripheral approach) of patients, a two-way MANOVA was performed. If the *p*-value is < 0.05, we reject the null hypothesis that there is no difference between the means and conclude that a significant difference does.

Propensity score (PS) matching was performed as previously reported (10). Briefly, the PSs were computed by binary logistic regression. A 1:1 nearest neighbor matching algorithm with a caliper of 0.1 of the standard deviation of the logit of the PS was chosen to achieve the highest possible representativeness and precision. Risk factors, which were statistically insignificant at baseline, were not considered as confounders and therefore not adjusted by PS matching. As 46 patients did not meet the matching criteria, they were discarded from the final analysis. Finally, the residual imbalances of covariates after matching were assessed by univariate tests, the Hansen–Bowers test and the relative multivariate imbalance measure.

## Results

Eighty-six patients met the inclusion criteria and were included in the analysis. A total of 58 patients (67%) required peripheral cannulation and 28 patients (33%) required central cannulation for VA-ECMO. The majority of patients underwent coronary artery bypass grafting (CABG) (52%). Other primary procedures were mostly combined CABG and valve surgery (29%). The mean age was 68 ± 10 years, and 64 of the patients (73%) were male. The VA-ECMO implantation for PCS took place in 43 patients (50%) in the operating room and 43 patients (50%) in the ICU. In central VA-ECMO group, the aortic cannula was inserted through a Dacron graft, and the chest was closed in 54% of the cases.

Seven (8.1%) patients received left ventricular (LV) venting, which was placed in the right superior pulmonary vein in 71.4%, in the LV apex in 14.3%, and in the pulmonary artery in 14.3% of cases.

Table 1 demonstrates the patient characteristics and demographics. There were no significant differences between groups except for higher EuroSCORE II (19 ± 14 vs. 11 ± 10, *p* = 0.007) and longer CBP time (229 ± 57 vs.180 ± 94, *p* = 0.01) in the central VA-ECMO group. Moreover, in a greater number of patients was chest left open after surgery in the central cannulation group (11, 39%) than that in the peripheral cannulation group (3, 5%) (*p* < 0.001).

**Table 1:** Patient characteristics and demographics.

	**Central (*N* = 28) (*n*, %) Mean ± SD**	**Peripheral (*N* = 58) (*n*, %) Mean ± SD**	***P*-value**
Age (years)	67 ± 11	69 ± 10	0.540
Body mass index	27 ± 7	27 ± 5	0.606
Male (*n*, %)	17 (61)	46 (79)	0.076
EuroSCORE II	19 ± 14	11 ± 10	0.007
X-Clamp time (min)	115 ± 48	88 ± 48	0.054
CPB time (min)	229 ± 57	180 ± 94	0.010
VA-ECMO duration (days)	7 ± 7	7 ± 5	0.926
LVEF < 30%	13 (46)	17 (29)	0.150
DM	10 (36)	25 (43)	0.641
AF	9 (32)	18 (31)	1.000
Elective procedure	10 (36)	21 (36)	1.000
Immediate intraoperative VA-ECMO	16 (57)	27 (47)	0.490
Chest left open after surgery	11 (39)	3 (5)	<0.001
IABP	11 (39)	28 (48)	0.493
LV venting	3 (11)	4 (7)	0.678
**Primary surgery**			
CABG	12 (43)	33 (57)	0.255
CABG + AVR	4 (14)	5 (9)	0.465
CABG + MVR ± TVR	5 (18)	11 (19)	1.000
AVR	0 (0)	6 (10)	0.171
Other procedures	7 (25)	3 (5)	0.012
Previous cardiac surgery	5 (18)	7 (12)	0.468

*CPB, cardiopulmonary bypass; LVEF, left ventricular ejection fraction; IABP, intra-aortic balloon pump; DM, diabetes mellitus; AF, atrial fibrillation; VA-ECMO, veno-arterial extracorporeal membrane oxygenation; CABG, coronary artery bypass grafting; AVR, aortic valve replacement; MVR, mitral valve replacement; TVR, tricuspid valve repair*.

Table 2 summarizes the outcome after VA-ECMO implantation. There was no significant difference in any of the postoperative parameters except for a significant higher in-hospital mortality rate in the central VA-ECMO group (79 vs. 52%, *p* = 0.02). Moreover, there was a non-significant trend toward a higher rate of weaning in the peripheral VA-ECMO group (29 vs. 52%, *p* = 0.063). There was no statistically significant difference in the resternotomy rates for bleeding between the central and the peripheral group (46 vs. 43%, respectively, *p* = 0.819).

**Table 2:** Outcome after VA-ECMO implantation.

	**Central (*N* = 28) (*n*, %) Mean ± SD**	**Peripheral (*N* = 58) (*n*, %) Mean ± SD**	***P*-value**
Chest tube outcome in first 24 h	1,251 ± 730	1,075 ± 947	0.384
RBC units transfused during the stay	48 ± 27	40 ± 29	0.226
Resternotomy for bleeding	13 (46)	25 (43)	0.819
Postoperative new-onset renal dialysis	19 (68)	39 (67)	1.000
Postoperative liver failure	9 (32)	16 (28)	0.800
Postoperative neurological injury	4 (14)	7 (12)	0.743
Postoperative GI complications	2 (7)	7 (12)	0.712
Weaning from VA-ECMO	8 (29)	30 (52)	0.063
ICU stay (days)	16 ± 15	19 ± 16	0.471
In-hospital mortality	22 (79)	30 (52)	0.020
Peripheral vascular complications	3 (11)	16 (28)	0.100

*RBC, red blood cell; GI, gastrointestinal*.

**Figure 1:**
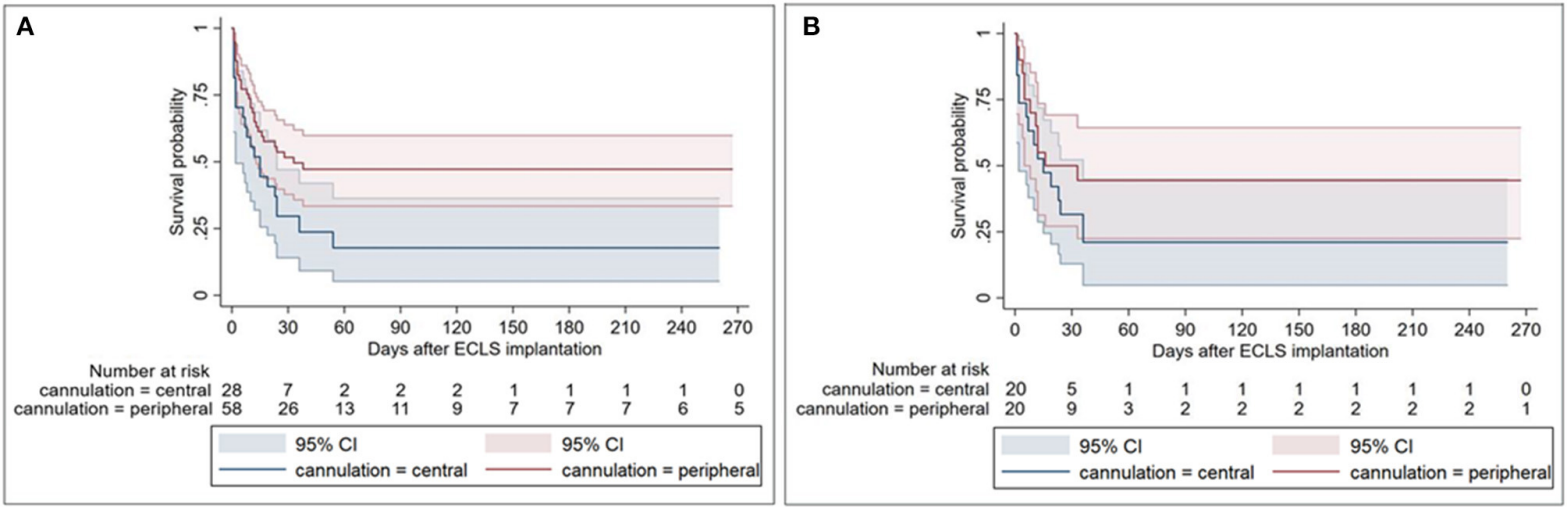
Kaplan–Meier survival curves depicting impaired survival in central compared to peripheral cannulation for postcardiotomy extracorporeal life support (ECLS) **(A)**, which attenuates after risk adjustment by propensity score matching **(B)**. The survival difference occurs early after surgery and is maintained in the later postoperative course.

Due to the fact that the groups were not identical, we decided to do a 1:1 PS matching to identify two matched groups. The following factors were included in the matching: EuroSCORE II, cross-clamp time, and type of the cardiac procedure. The PS analysis resulted in 20 patients remaining in each group (Table 3). Table 4 shows the difference in the postimplantation parameters between both groups after PS matching. Interestingly, no significant differences in postoperative bleeding (1,219 ± 651 vs. 1,143 ± 1,317 ml, *p* = 0.824), transfusion (48 ± 29 vs. 45 ± 31, *p* = 0.755), duration of ICU stay (16 ± 14 days vs. 18 ± 19 days, *p* = 0.638), and in-hospital mortality (75 vs. 55%, *p* = 0.320) were observed between the matched groups. Furthermore, the rate of PV complications prior to and after matching remains similar between the groups (11 vs. 28% and 16 vs. 25%, *p* = 0.100 and *p* = 0.695, respectively). Figures 1A,B show the Kaplan–Meier survival curve in the unmatched and matched analyses.

**Table 3:** Patient characteristics and demographics after propensity score matching.

**Propensity score**	**Central (*N* = 20) (*n*, %) Mean ± SD**	**Peripheral (*N* = 20) (*n*, %) Mean ± SD**	***P*-value**
Age (years)	67 ± 10	60 ± 10	0.738
Body mass index	26 ± 7	27 ± 5	0.799
Male (*n*, %)	14 (70)	14 (70)	1.000
EuroSCORE	15 ± 10	16 ± 13	0.694
X-Clamp time (min)	126 ± 44	89 ± 53	0.062
CPB time (min)	228 ± 61	204 ± 108	0.448
VA-ECMO duration (days)	8 ± 8	7 ± 6	0.561
LVEF < 30%	10 (50)	6 (30)	0.333
DM	7 (35)	7 (35)	1.000
AF	7 (35)	5 (25)	0.731
Elective procedure	9 (45)	8 (40)	1.000
Immediate intraoperative VA-ECMO	10 (50)	9 (45)	1.000
Chest left open after surgery	8 (40)	1 (5)	0.020
IABP	8 (40)	6 (30)	0.741
LV venting	2 (10)	2 (10)	1.000
**Primary surgery**			
CABG	7 (35)	11 (55)	0.341
CABG + AKR	4 (20)	1 (5)	0.342
CABG + MKR ± TKR	5 (25)	3 (15)	0.695
AKR	0 (0)	3 (15)	0.231
Other procedures	4 (20)	2 (10)	0.661

*CPB, cardiopulmonary bypass; LVEF, left ventricular ejection fraction; IABP, intra-aortic balloon pump; DM, diabetes mellitus; AF, atrial fibrillation; VA-ECMO, veno-arterial extracorporeal membrane oxygenation; CABG, coronary artery bypass grafting; AVR, aortic valve replacement; MVR, mitral valve replacement; TVR, tricuspid valve repair*.

**Table 4:** Outcome after VA-ECMO implantation after propensity score matching.

	**Central (*N* = 20) (*n*, %) Mean ± SD**	**Peripheral (*N* = 20) (*n*, %) Mean ± SD**	***P*-value**
Chest tube outcome in first 24 h	1,219 ± 651	1,143 ± 1,317	0.824
RBC Units transfused during the stay	48 ± 29	45 ± 31	0.755
Resternotomy for bleeding	9 (45)	9 (45)	1.000
Postoperative new onset dialysis	14 (70)	15 (75)	1.000
Postoperative liver failure	6 (30)	8 (40)	0.741
Postoperative neurological injury	3 (15)	2 (10)	0.605
Postoperative GI complications	2 (11)	1 (5)	0.712
Weaning from VA-ECMO	5 (25)	9 (45)	0.320
ICU stay (days)	16 ± 14	18 ± 19	0.638
In-hospital mortality	15 (75)	11 (55)	0.320
Peripheral vascular complications	3 (16)	5 (25)	0.695

*RBC, red blood cell; GI, gastrointestinal*.

## Discussion

The main findings of this single-center study including 86 consecutive patients supported with VA-ECMO in a postcardiotomy setting can be summarized as follows:

1. In the unmatched group of patients, the central VA-ECMO group tends to have higher mortality rate after the index cardiac procedure.
2. The postimplantation morbidity and mortality remain similar between the groups after PS matching, highlighting the fact that none of the implantation technique is advantageous over the other.
3. The similar bleeding rates in the matched group may be related to chest closure in the majority of the central ECMO group.
4. The rates of PV complications are similar if distal leg perfusion is used in all patients.

The PCS is presumably an annihilating complication after cardiac surgical procedures and correlated with a soaring mortality rate. What seems to be the topmost choice for patients with refractory PCS is the VA-ECMO implantation. The ideal cannulation approach (central vs. peripheral) for PCS is yet to be defined. It was therefore the aim of this study to shed light on the unanswered question in the postcardiotomy setting.

The utilization of VA-ECMO has been increasing during the last decades, and PCS constitutes one of the most common indications (1, 11–14). Although it is considered an ultimate option, the use of VA-ECMO has gradually reduced in-hospital mortality over time as well as remained a resource-consuming treatment (12–14). Despite growing worldwide experience, the overall survival to hospital discharge was 41.4% in adults in a current Extracorporeal Life Support Organization (ELSO) Registry Report (5). Therefore, there are some concerns arising about costs, benefits, and ethics.

Central and peripheral cannulation strategies are both to be utilized habitually on a PCS clinical scenario. The VA-ECMO implantation for PCS according our results took place in 43 patients (50%) in the operating room and 43 patients (50%) in the ICU, as reported in the results of this study. In case of a PCS scenario, failure from CPB weaning regularly requires the implementation of VA-ECMO and usually a central configuration can easily be inaugurated utilizing the already placed cannulas for the previous CPB. A peripheral access can be achieved percutaneously using the femoral or, less frequently, axillary or subclavian (9, 15) artery and femoral or jugular vein (6, 8). Sorokin et al. (3) reported previously the details on appropriate configuration and cannulation strategy for ECMO.

There are both benefits and pitfalls of each cannulation strategy (16): the central cannulation ensures an antegrade flow, which may provide a better LV unloading. The peripheral one directs a retrograde flow toward the aortic valve and causes an increase in LV afterload. Moreover, it is a fundamental issue that the peripheral cannulation leads to Harlequin syndrome. On the other hand, it is a less time-consuming and less invasive technique, which allows sternal closure. Central VA-ECMO might also be initiated with the closed chest in PCS. A Dacron graft can be anastomosed to the ascending aorta, which may be tunneled to exit at the subxiphoid region, allowing patients extubation and mobilization after surgery in case of prolonged support or bridge to destination therapy. However, a potential compression of the graft along its course through the mediastinum toward the subxiphoid exit points may cause an insufficient hemodynamic support. Another possible outlet for the cannulae in closed-chest conditions may be directly through the cranial end of the sternotomy wound. This may avoid a possible cardiac compression by cannulae along their course through the mediastinum.

Mariscalco et al. (17) compared peripheral and central VA-ECMO in a retrospective study of 781 patients with PCS at 19 cardiac surgery centers. This multicenter study showed that central cannulation was associated with greater in-hospital mortality than peripheral cannulation (17). Although our unmatched data support this finding, after PS matching, complication rates and outcome were similar regardless of the cannulation strategy.

The subclavian artery cannulation should provide several advantages by allowing to mimic the blood flow of the central cannulation approach in contrast to femoral artery (9, 18). The advantages include the lack of atherosclerosis, minimizing atherosclerotic embolization, and preferential delivery of oxygenated blood to the heart and brain (19). Therefore, the subclavian approach appears advisable in patients with peripheral arterial occlusive disease because of its lack of atherosclerosis in comparison to the femoral artery. Ranney et al. (9) reported a higher rate of vascular complications (particularly fasciotomy and amputation) and bleeding at the cannulation site (37.5, 30.6, and 13.9%, respectively). In that study, a trend toward a higher incidence of cerebrovascular events was also observed (9). We believe that subclavian cannulation is advantageous when longer support duration is anticipated to allow patients' extubation and mobilization.

The hemodynamic effects and end-organ function regarding cannulation approach is not well-described in the literature. Our group (2) compared the immediate trends in hemodynamics, oxygenation, ventilation, and end-organ function of patients on either peripheral or central VA-ECMO support. No particular advantage of one technique over the other was observed. The course of serum lactate levels under ECMO plays a predictive role in 30-day mortality (20, 21). However, there were no differences between peripheral and central cannulation regarding the mean peak lactate level as a marker of tissue perfusion and end-organ damage (7). In a series of 517 patients reported by Rastan et al. (6), lactate level > 10 mmol/L immediately after ECMO implantation was a significant predictor of mortality. Persistent lactate values > 10 mmol/L were also associated with increased mortality (6). They also found that arterial cannulation site did not significantly influence hospital outcome, but percutaneous venous femoral cannulation was associated with adverse outcomes (6).

Supporting an impaired ventricle with ECMO may lead to LV overload, especially in peripheral configuration due to retrograde flow toward the LV, causing an increased afterload (22). The potential consequences of LV overload are LV dilatation, increased left atrial pressure, blood stasis, and thrombus formation in cardiac chambers and pulmonary edema (22). Despite being adopted in the minority of patients, LV venting is of paramount importance during PCS. However, the optimal method for LV venting is still unclear. Central configuration allows to place an additional cannula in the LV through the right superior pulmonary vein or LV apex. On the other hand, peripheral VA-ECMO in closed-chest conditions may need another method. Intra-aortic balloon pump (IABP), although controversial (22, 23), is still being widely used in clinical practice. In some PCS series, the non-use of IABP was associated with a trend to worse survival (6, 24), whereas the others did not find any differences in survival outcomes (25, 26). Alternative techniques for percutaneous LV venting include Impella® (ABIOMED Inc., Danvers, Massachusetts) or pulmonary artery venting (22, 25, 26). The optimal combination of either peripheral or central cannulation and venting methods needs further research.

Beside its life-saving effect, complications of VA-ECMO are numerous and impair the overall outcomes inevitably (6, 11). Our single-center experience does not favor central or peripheral cannulation in terms of reoperation for bleeding and number of transfused RBC units. Regardless of cannulation strategy, bleeding, transfusion, and revision for bleeding constitute major problems on VA-ECMO (6). Recently, Djordjevic et al. (27) reported a reexploration rate of 93% of all patients on central VA-ECMO. Central cannulation is opted for by virtue of the following: to leave the chest open to avoid tamponade as well as to allow cardiac edema to resolve, to inherit the previously inserted cannulae for ECMO circuit, and to avoid limb ischemia due to femoral artery cannulation. We expected to see more bleeding complications in the central VA-ECMO group. However, our data support the fact that the bleeding issue in the postcardiotomy setting may be rather derived by the ECMO-related bleeding tendency than the surgical technique implantation. Furthermore, another explanation may be the fact that we tend to use a prosthesis in the majority of central VA-ECMO patients to facilitate chest closure (2). Therefore, the bleeding rate was not significantly higher in the central VA-ECMO group because bleeding from sternal edges was precluded.

The present study showed that PV complications in the peripheral VA-ECMO group exceeded that of the central VA-ECMO group prior to and after matching; however, interestingly, this finding did not reach statistical significance. The main explanation of this finding is the fact that the femoral vein was frequently used as inflow cannula also for central VA-ECMO group and a distal leg perfusion catheter was used in the peripheral VA-ECMO group to avoid limb ischemia. In our study, the majority of the implantation (58.6%) was percutaneous. Loforte et al. (28) showed that central cannulation in PCS resulted in increased bleeding and continuous VV hemofiltration rates compared to peripheral access (62.7 vs. 48.4% and 56.8 vs. 43.6%, respectively). Ko et al. (8) investigated a higher rate of neurologic complication with open femoral ECMO. However, after matching the groups, no significant differences in these morbidities were observed in the present study.

### Limitations

The main limitation of this study is its retrospective single-center nature. However, the majority of the data were already prospectively collected in the hospital databank. Moreover, the implantation approach was not randomized, and the decision regarding central vs. peripheral cannulation was at the discretion of the implanting surgeon in the operating room. However, ECMO implantations in the ICU were performed exclusively peripherally at the bed site. Furthermore, no hemodynamic data or data on vasopressor requirement were available to compare between the groups. After PS matching, a large number of patients were discarded from the analysis, which may potentially influence the results.

## Conclusion

This study of a matched group of patients using central vs. peripheral VA-ECMO for postcardiotomy patients showed no advantage of one approach over the other. The high rate of chest closure in the central VA-ECMO group and the exclusive implication of the distal leg perfusion catheter may explain this finding. Decision-making for the cannulation strategy should be individualized and adjusted to the clinical scenario. Further randomized studies are necessary to identify the ideal cannulation strategy in the PCS population.

## Data Availability Statement

The raw data supporting the conclusions of this article will be made available by the authors, without undue reservation.

## Ethics Statement

The studies involving human participants were reviewed and approved by Ethic Committee of University Hospital of Düsseldorf. Written informed consent for participation was not required for this study in accordance with the national legislation and the institutional requirements.

## Author's Note

Presented in part at the 32nd EACTS Annual Meeting in Milan, Italy.

## Author Contributions

NK: conceptualization, data curation, formal analysis, investigation, methodology, and writing—original draft. NS: data curation, supervision, writing—original draft, writing—review and editing, and project administration. GP: formal analysis and writing—review and editing. HA, PA, AA, RW, and AL: writing—review and editing. DS: conceptualization, methodology, supervision, writing—original draft, and writing—review and editing. All authors contributed to the article and approved the submitted version.

## Conflict of Interest

The authors declare that the research was conducted in the absence of any commercial or financial relationships that could be construed as a potential conflict of interest.

## Publisher's Note

All claims expressed in this article are solely those of the authors and do not necessarily represent those of their affiliated organizations, or those of the publisher, the editors and the reviewers. Any product that may be evaluated in this article, or claim that may be made by its manufacturer, is not guaranteed or endorsed by the publisher.

## Abbreviations

VA-ECMO: veno-arterial extracorporeal membrane oxygenation
PCS: postcardiotomy cardiogenic shock
PV: peripheral vascular
GI: gastrointestinal
PS: propensity score.
